# Machine Learning and Feature Selection Applied to SEER Data to Reliably Assess Thyroid Cancer Prognosis

**DOI:** 10.1038/s41598-020-62023-w

**Published:** 2020-03-20

**Authors:** Moustafa Mourad, Sami Moubayed, Aaron Dezube, Youssef Mourad, Kyle Park, Albertina Torreblanca-Zanca, José S. Torrecilla, John C. Cancilla, Jiwu Wang

**Affiliations:** 1 Division of Otolaryngology–Head & Neck Surgery, Jamaica Hospital Medical Center, New York, NY USA; 2 Department of Otolaryngology-Head and Neck Surgery, University of Montreal, Montreal, Canada; 3 Department of General Surgery, Tufts University, Boston, MA USA; 4 Jagiellonian University, Krakow, Poland; 5 Comprehensive Tissue Centre, Alberta Health Services, Alberta, Canada; 6 Department of Neurosciences, Center for Research in Biological Systems, University of California, San Diego, School of Medicine, La Jolla, CA USA; 7 Departamento de Ingeniería Química, Facultad de Ciencias Químicas, Universidad Complutense de Madrid, Madrid, Spain; 8 Scintillon Institute, San Diego, CA USA

**Keywords:** Disease-free survival, Targeted therapies

## Abstract

Utilizing historical clinical datasets to guide future treatment choices is beneficial for patients and physicians. Machine learning and feature selection algorithms (namely, Fisher’s discriminant ratio, Kruskal-Wallis’ analysis, and Relief-F) have been combined in this research to analyse a SEER database containing clinical features from de-identified thyroid cancer patients. The data covered 34 unique clinical variables such as patients’ age at diagnosis or information regarding lymph nodes, which were employed to build various novel classifiers to distinguish patients that lived for over 10 years since diagnosis, from those who did not survive at least five years. By properly optimizing supervised neural networks, specifically multilayer perceptrons, using data from large groups of thyroid cancer patients (between 6,756 and 20,344 for different models), we demonstrate that unspecialized and existing medical recording can be reliably turned into power of prediction to help doctors make informed and optimized treatment decisions, as distinguishing patients in terms of prognosis has been achieved with 94.5% accuracy. We also envisage the potential of applying our machine learning strategy to other diseases and purposes such as in designing clinical trials for unmasking the maximum benefits and minimizing risks associated with new drug candidates on given populations.

## Introduction

Machine learning as algorithmic advancement in the past few years dramatically improved our range of potential implementation of artificial intelligence for tasks such as learning and playing the Go game, environment feature recognition for self-driving, and in medical applications^1,2^. Within the machine learning scope, artificial neural networks (ANNs) are a set of algorithms that recognize patterns and learn from inputs and outputs to make useful connections without pre-set rules^3^. Furthermore, ANNs and their performance correlate well with the training data size and are more adept at pattern recognition and classification when analysing large hospital records than traditional statistical modelling applied in some of the more recent cancer prognostication applications^4,5^. ANN models are designed in layers to learn increasingly higher-dimension and remote representations of the input data and devise meaningful outcomes to feed the next layer.

In this work, we tested three separate neural network models to determine the outcomes of thyroid cancer patients after diagnosis from distilling the U.S. Surveillance Epidemiology and End Results (SEER) database. Although back in 2015 thyroid cancer cases in the United States were predicted to increase to 92,000 by 2020^6^, and current estimates indicate that in 2019 around 52,000 are projected instead, these numbers still signify that thyroid cancer incidence rates continue to increase^7^. Specifically, regarding women, thyroid cancer ranks sixth compared to other types of cancer in terms of incidence with almost 38,000 new estimated cases per year^7^. These trends can be mainly attributed to an increase in incidence of well differentiated thyroid cancers (WDTC) and may be in part due to the increasing use of neck ultrasonography or other imaging modalities leading to early diagnosis and treatment^8^. The steady rise in incidence of thyroid cancer prompts the development of improved methodologies for accurate tumour staging and prognostication to guide treatment and predict survival.

In this line, recent research has revealed the existence of potential biomarkers that show the ability to aid in thyroid cancer prognosis prediction including proteins, DNA copy number amplifications (CNAs), and non-coding RNA, such as glycoprotein Wnt inhibitor dickkopf-1, CNAs of LINC01061, and ZFAS1, respectively^9–11^. On the other hand, the *Manual for Staging of Cancer* by the American Joint Committee on Cancer Staging (AJCC) states that a “classification scheme for cancer must encompass all attributes of the tumour that define its life history”^12^. Modern day cancer staging is largely based on clinical criteria used to model and predict tumour prognostication. The most commonly utilized staging schema for WDTC is the TNM system that utilizes tumour size (T), nodal status (N), and presence/absence of metastatic disease (M). Other prognostic indices include the AMES (Age, Metastases, Extent, and Size) and MACIS (Metastasis, Age, Completeness of resection, local Invasion, and Size)^13,14^. These described indices are largely based on retrospective clinical data that utilize univariate and multivariate statistical analysis.

Our study design was based on the reasoning that recent advancements in machine learning have provided opportunities to uncover variable relationships otherwise inaccessible through other more common statistical approaches in modelling datasets like the thyroid cancer records within the U.S. SEER database. Our study has led to the most accurate method to date utilized to predict thyroid cancer survival using data compiled from the SEER program registry. We validated our network through a direct comparison to an ANN generated using the AJCC TNM staging system, further demonstrating the power of our artificial intelligence system when coupled with relevant clinical features. Consequently, we believe our findings reveal the need for change in current thyroid cancer assessment standards, coinciding with new studies in the field^15^.

## Results

### Database and artificial neural networks used in this study

During this research, non-linear algorithms known as multilayer perceptrons (MLPs; in our case consisting of 3 layers: an input layer, a hidden layer, and an output layer) have been employed to interpret the databases^16^. In total, 25,063 thyroid cancer entries were extracted from the initial SEER database that met the inclusion criteria set for the novel models (MLP-1 and MLP-2). Relevant demographic and clinical data regarding these patients are shown in Table 1, which is subdivided into patients who survived more than ten years since diagnosis (alive) and those who passed away within the first five years due to the disease (cause of death thyroid cancer; COD-TC). Within the employed database, several thyroid cancer risk factors are covered including gender, as there are three times more women patients than men, and age, where risk peaks vary depending on gender (in their 40 s and 50 s for women, 60 s and 70 s for men). On the other hand, certain hereditary conditions are also risk factors, but to a lesser extent as most thyroid cancer patients do not develop the disease due to inheritance or even have affected family members (not covered in the database used). Other risk factors include diets with low iodine content, exposure to radiation, and even height and weight (data not recorded)^17^.

**Table 1:** Demographic and clinical information regarding the 25,063 thyroid cancer patients that met the requirements for the main modelling phase.

Patients	Alive				COD-TC		
Number of participants (% of total)	24,025 (95.9%)				1,038 (4.1%)		
Gender (male/female; % of each)	4,896/19,129 (20.4%/79.6%)				426/612 (41.0%/59.0%)		
Race (white/black/American Indian, Alaska Native, Asian, Pacific Islander/unknown; % of each)	19,774/1,295/2,777/179 (82.3%/5.4%/11.6%/0.7%)				829/70/137/2 (79.9%/6.7%/13.2%/0.2%)		
Grade (I (well differentiated)/II (moderately differentiated)/III (poorly differentiated))	3,671/1,143/175 (grades for remaining cases were unrecorded)				75/69/189 (grades for remaining cases were unrecorded)		
Age ± standard deviation	40.5 ± 12.8				65.9 ± 13.4		
Age groups (top); proportion of each group (Alive/COD-TC) in % (bottom)	20–29	30–39	40–49	50–59	60–69	70–79	≥80
	16/0.8	28/1.8	27/7.3	17/17	7.2/22	2.9/29	0.3/22

The data entries corresponding to thyroid cancer patients were used to train and validate three different MLPs differing in terms of independent variables (*vide infra*) and number of available samples (Table 2), as not all variables were available or registered for every patient (as soon as one of the employed independent variables was incomplete, the corresponding sample was removed; the variables employed, original names, their values after pre-processing, legend, and missing rates are included in the Supplementary Information section (Excel sheet: “Database of Variables Used”)). Regarding the designed, optimized, and validated MLPs, the final selected functions and optimized network architectures and parameters can be seen in Table 2 (further explained in Materials and Methods).

**Table 2:** Selected functions, optimized parameters, MLP architecture, as well as data points employed during the design of MLP-1, MLP-2, and MLP-3.

	MLP-1	MLP-2	MLP-3
Training function	Levenberg-Marquardt backpropagation		
Transfer function	Sigmoid		
Number of data points (Alive/COD-TC)	8,477 (8,256/221)	20,344 (19,848/496)	6,756 (6,515/241)
Input nodes	7	3	3
Hidden neurons	19	18	4
Output neurons	1 (all binary classifiers)		
Learning coefficient (Lc)	0.001	0.001	0.5005
Lc-decrease	1	0.001	0.5005
Lc-increase	100	100	51

### MLP-1 – seven independent variables

**Figure 1:**
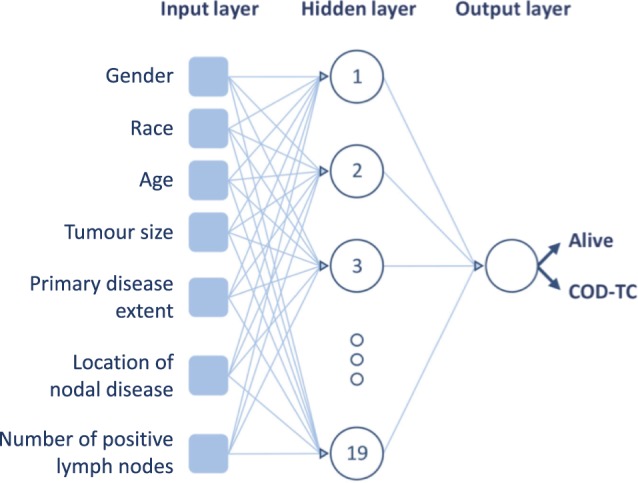
Architecture of MLP-1. The independent variables, number of hidden neurons, and output are shown.

The independent variables employed to train this model were age, race, gender, tumour size, primary disease extent, location of nodal disease, and number of positive lymph nodes (Fig. 1). In total, 8,477 entries were available for this model (Table 2) and were subsequently used to train MLP-1 to classify cases into alive or thyroid-related death. In total, the network was able to correctly estimate 94.49% of outcomes (correct hits divided by total data points) when applied to entries with blinded classes. The accuracies are reported with confidence intervals (CIs) of 95% in Table 3, which were calculated following Eq. (1).

1 $$int=z\sqrt{(acc(1-acc))/n)}$$

where ***int*** represents the radius of the CI, ***z*** is the number of standard deviations from the Gaussian distribution (1.96 in this case, to reach 95% confidence), ***acc*** is the reported accuracy of a given model, and ***n*** symbolizes the amount of data points from the test datasets evaluated.

**Table 3:** Statistical results of MLP-1, MLP-2, and MLP-3 for their independent test datasets (n = 3). Accuracy, specificity × 100, and sensitivity × 100 reported with 95% confidence interval radius (CIR).

	MLP-1	MLP-2	MLP-3
Accuracy ± 95% CIR (%)	94.49 ± 0.88	91.09 ± 0.71	80.87 ± 1.71
Alive (Specificity × 100) ± 95% CIR (%)	94.45 ± 0.90	91.08 ± 0.72	80.84 ± 1.75
COD-TC (Sensitivity × 100) ± 95% CIR (%)	96.36 ± 4.95	91.41 ± 4.86	81.40 ± 8.22
Threshold	0.0447	0.028	0.0319
MCC	0.501	0.383	0.304
PPV	0.277	0.180	0.158
NPV	0.999	0.998	0.990
F1 Score	0.431	0.301	0.265

In predicting alive cases, the network was 94.45% accurate (correct alive cases divided by total alive cases; specificity × 100), with 96.36% accuracy in predicting thyroid-related deaths (correct COD-TC cases divided by total COD-TC cases; sensitivity × 100). CIs of 95% have also been calculated for these two metrics via Eq. (1), by changing **acc** for the respective values of specificity × 100 or sensitivity × 100 (Table 3)^18^. The presented results or model performances were reached after using an independent and randomly separated test dataset, which contains “blinded” samples that have never been seen by the optimized MLP. Furthermore, in order to define the threshold of all of our models (to decide which predicted result is considered as an alive or COD-TC case), our main criterion was to reach comparable specificity and sensitivity values while giving priority to sensitivity, as identifying cases with worse prognosis is a critical point of the algorithms (low false negative rate sought). In other words, the threshold was set at the exact point where sensitivity surpassed specificity.

**Figure 2:**
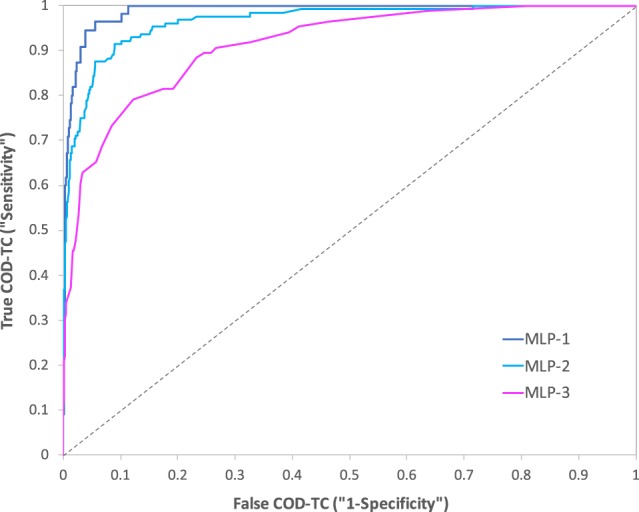
ROC curves regarding the binary classifiers MLP-1 (AUC = 0.988), MLP-2 (AUC = 0.966), and MLP-3 (AUC = 0.914). Baseline shown with discontinuous line (“AUC = 0.500”). These curves with 95% CIs can be seen in the Supplementary Information section (Excel sheet: “ROC Curves”).

These results led to a receiver operating characteristic (ROC) curve with a notably large area under the curve (AUC) of 0.988 (Fig. 2; in Supplementary Information section shown with 95% CIs (Excel sheet: “ROC Curves”)). Other standard metrics including Matthews correlation coefficient (MCC; known for being a suitable parameter to handle unbalanced data, as is the case (*vide infra*), leading to values ranging from −1, absolute disagreement between prediction and real observation, to +1, perfect prediction, where 0 means random prediction^19^), precision or positive predictive value (PPV), negative predictive value (NPV), and F1 score (harmonic mean of PPV and sensitivity) are also shown in Table 3. As can be noticed, all the metrics reveal solid results except for the precision (and related F1 score) or, in other words, the percentage of true positives among all those classified as positives. This a direct reflection of the unbalanced nature or low prevalence found in the database (24,025 alive versus 1,038 COD-TC cases (Table 1); 4% prevalence) and the fact that the threshold has been set to prioritize the correct classification of thyroid cancer patients with poor prognosis, i.e. the COD-TC group, which shows very high sensitivity (96.4 ± 5.0%, Table 3).

### MLP-2 – three independent variables via feature selection

In order to identify the three variables that contain the strongest prediction power (exactly three variables were selected to employ the same amount as the TNM model, thus reaching a comparable MLP architecture; *vide infra*), a second MLP model was trained. Three different filter-based feature selection (FS) algorithms (Fisher’s discriminant ratio^20^, Kruskal-Wallis test^21^, and Relief-F^22^), which rank variables according to their discriminative power for a successive task (further detailed in Materials and Methods section)^23^, were used on the features of the initial database. The functions which represent each of the filter-based FS methods are shown in Eqs. (2–4). Fisher’s discriminant ratio (FDR; Eq. (2)) uses linear calculations to determine the discriminative power of a variable. It operates by searching for a line that can separate the data samples into their corresponding classes the best way possible^20^.

2 $$FDR=\,\frac{{((\overline{{x}_{1}})-(\overline{{x}_{2}}))}^{2}}{Var({x}_{1})+Var({x}_{2})}$$

where $\overline{{{\boldsymbol{x}}}_{1}}$ and $\overline{{{\boldsymbol{x}}}_{2}}$ represent the means of the values of a certain feature for classes ***x***_***1***_ and ***x***_***2***_, respectively, while ***Var(x***_***1***_***)*** and ***Var(x***_***2***_***)*** are the variances of these datasets.

The Kruskal-Wallis test (KW; Eq. (3)) relies on non-parametric calculations to rank features by comparing the medians of the different classes. It is able to interpret non-linear relations between the values of the variable evaluated and the class label and determines whether the medians of the values of a feature of two or more classes are equal or not to rank them in terms of discriminative capability^21^.

3 $$KW=\frac{12}{N(N+1)}\sum _{i=1}{n}_{i}{({\bar{r}}_{i})}^{2}-3\left(N+1\right)$$

where ***N*** is the amount of observations or samples in all the groups, ***n***_***i***_ is the number of observations in group ***i***, and ${\bar{{\boldsymbol{r}}}}_{{\boldsymbol{i}}}$ represents the mean of the ranks of observations in group ***i***.

Finally, the Relief-F algorithm (R_F_; Eq. (4)) is based on evaluating features by the extent of their ability to distinguish the values of instances or samples that are near to each other. When analysing a sample value, it seeks for the nearest neighbours, one per class (same and different), and adjusts the feature weighting vector to enable ranking variables according to their ability to discriminate neighbour samples from others corresponding to different classes^22^.

4 $${R}_{F}\left({f}_{i}\right)=\frac{1}{2}\sum _{t=1}d\left({f}_{t,i}-{f}_{NM({x}_{t}),i}\right)-d\left({f}_{t,i}-{f}_{NH({x}_{t}),i}\right)$$

where ***f***_***t***,***i***_ represents the value of the sample analysed (***x***_***t***_) of a specific feature (***f***_***i***_), while ***f***_***NM(xt)***,***i***_ and ***f***_***NH(xt)***,***i***_ are the values of the ***i***^***th***^ feature corresponding to the nearest neighbours of different and same classes, respectively. Finally, ***d(·)*** is the function employed as a distance measurement between the sample and the nearest neighbours.

**Figure 3:**
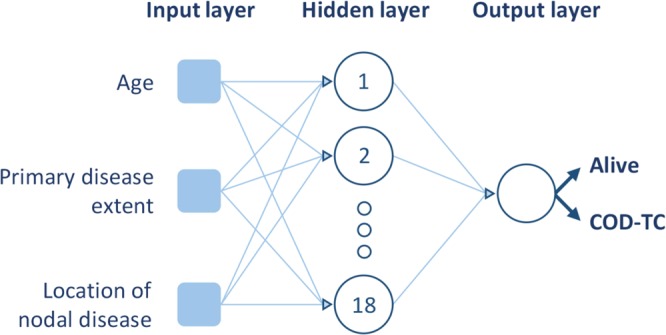
Architecture of MLP-2. The selected independent variables (through FS algorithms), number of hidden neurons, and output are shown.

Considering the results provided by the three presented FS methods^20–22^, the variables were ranked from most relevant to least, in terms of classifying power (Table 4). The scores provided by the FS algorithms for every variable are shown in the Supplementary Information section (Excel sheet: “FS Scores”). We found that the most predictive variables from MLP-1 were age, location of nodal disease, and primary disease extent. The number of positive lymph nodes, race, tumour size, and gender were identified as variables with less predictive value and were not included. The model was subsequently reduced to three independent variables (Fig. 3). In total, 20,344 entries were used to train MLP-2 (Table 2). The network had an overall accuracy of 91.09%, predicting 91.08% of alive cases, and 91.41% of thyroid-related death (Table 3), and its ROC curve revealed a very high AUC of 0.966 (Fig. 2; in Supplementary Information section shown with 95% CIs (Excel sheet: “ROC Curves”)). MCC, PPV, NPV, and F1 score are also shown for MLP-2 in Table 3.

**Table 4:** Results of the three feature selection processes carried out. The variables are ranked from left to right in terms of discriminative power according to each algorithm. Variables are labelled as: (1) gender, (2) race, (3) age, (4) tumour size, (5) primary disease extent, (6) location of nodal disease, and (7) number of positive lymph nodes. Variables 3, 5, and 6 are the overall highest ranked clinical variables.

Feature selection algorithm	Ranking
Fisher’s discriminant ratio	3, 5, 4, 6, 1, 7, 2
Kruskal-Wallis test	5, 6, all others
Relief-F	3, 7, 6, 2, 4, 5, 1

### MLP-3 – TNM model

**Figure 4:**
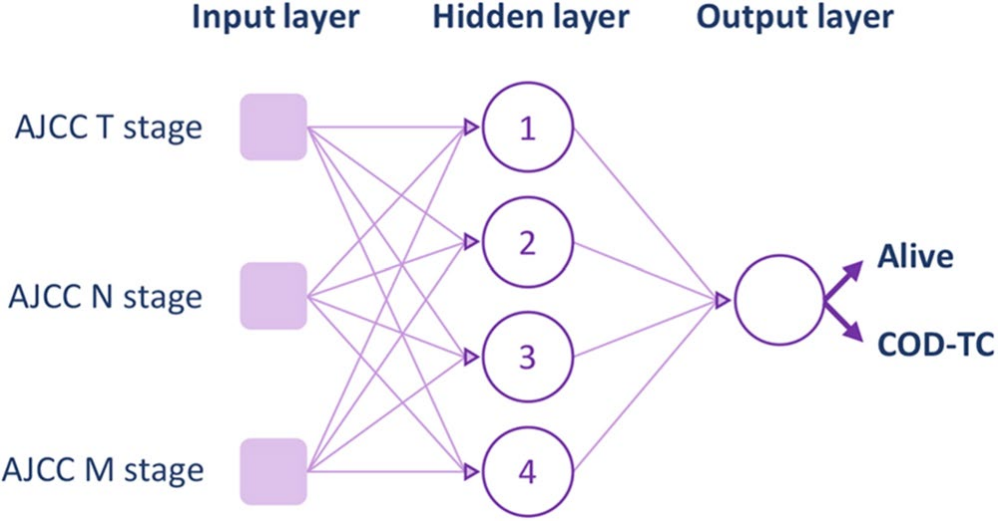
Architecture of MLP-3. The independent variables (TNM), number of hidden neurons, and output are shown.

Finally, a third model was designed only using the variables that are based on the TNM staging system (tumour size (T), number of positive nodes (N), and presence of metastases (M)) established by the AJCC (Fig. 4). In total, 6,756 entries were used to train the network (Table 2), and it must be noted that the range of the group of alive patients (good prognosis) was changed from 10 to 7.5 years survived since diagnosis due to data being unavailable concerning the three independent variables (T, N, and M) inevitably needed to train this model (see Supplementary Information section (Excel sheet: “Database of Variables Used”)). The overall network accuracy was seen reduced to 80.87%, correctly identifying 80.84% of alive cases, and 81.40% of thyroid-related deaths (Table 3), leading to a ROC curve with an AUC of 0.914 (Fig. 2; in Supplementary Information section shown with 95% CIs (Excel sheet: “ROC Curves”)), agreeing with the FS process results, which reveal the weaker relevance of these variables used by the AJCC. Values regarding MCC, PPV, NPV, and F1 score are also shown for MLP-3 in Table 3. Comparing the results of the three MLPs illustrates the usefulness of filter-based FS algorithms, as well as their strength when combined with machine learning-based models like ANNs.

The labels (alive and COD-TC) and predictions regarding the randomized test datasets (in triplicate) for the three MLPs and three PLS-DAs (partial least squares-discriminant analysis, see below) are shown in the Supplementary Information section, as well as true positives and negatives, and false positives and negatives for each model (Excel sheets: “MLP Predictions” and “PLS-DA Predictions”). These tests were carried out three times in order to further validate the reproducibility and plasticity or flexibility of the MLPs.

### Partial least squares-discriminant analysis models

In order to further validate the use of machine learning-based models for predicting thyroid cancer patient outcomes, we then compared our results with those generated by three analogous partial least squares-discriminant analysis (PLS-DA) models, which represent a classic mathematical approach based on the creation of linear regressions to estimate categorical variables (alive and COD-TC in this scenario). They have been developed with the same sets of independent variables (PLS-DA-1 is comparable to MLP-1, and so on). The same validation strategy has been employed (10% of randomized samples were used to test the linear regressions three different times; analogous to the procedure used for the MLPs). The results provided by these linear models can be seen in Table 5, revealing a weaker performance when compared to their corresponding MLPs (Table 3; a quantitative comparison is shown in the following subsection). Also, their ROC curves, with AUCs of 0.963, 0.958, and 0.885 for PLS-DA-1, PLS-DA-2, and PLS-DA-3, respectively, are shown in the Supplementary Information section with 95% CIs (Excel sheet: “ROC Curves”).

**Table 5:** Statistical results of PLS-DA-1, PLS-DA-2, and PLS-DA-3 for their independent test datasets (n = 3). Accuracy, specificity × 100, and sensitivity × 100 reported with 95% confidence interval radius (CIR).

	PLS-DA-1	PLS-DA-2	PLS-DA-3
Accuracy ± 95% CIR (%)	87.16 ± 1.30	89.27 ± 0.78	78.80 ± 1.78
Alive (Specificity × 100) ± 95% CIR (%)	87.05 ± 1.32	89.23 ± 0.78	78.71 ± 1.81
COD-TC (Sensitivity × 100) ± 95% CIR (%)	91.18 ± 6.74	91.13 ± 5.00	81.54 ± 9.43
Threshold	0.086268	0.102	0.113
MCC	0.353	0.344	0.251
PPV	0.162	0.149	0.112
NPV	0.997	0.998	0.992
F1 Score	0.275	0.256	0.198

### Comparing results of the classifiers

Firstly, we wanted to analyse the effect of the different variables on the performance of the MLP classifiers. As can be seen in the ROC curves (Fig. 2; in Supplementary Information section shown with 95% CIs (Excel sheet: “ROC Curves”)) and statistical performance (Table 3), the classifiers trained with new independent variables (MLP-1 and MLP-2), different to the standard TNM ones (MLP-3), are more reliable and accurate (94.49 ± 0.88% and 91.09 ± 0.71% versus 80.87 ± 1.71%, respectively (narrow 95% CIs further strengthen the results); AUCs from ROC curves of 0.988 and 0.966 versus 0.914, respectively), revealing the stronger prognostic power of these medical attributes. It is worth mentioning that MLP-2, although slightly weaker than MLP-1, possesses the same number of variables as the TNM-model (three variables) and yet vastly outperforms it (over 10% better accuracy, as well as improved sensitivity and specificity). Nevertheless, due to the availability of such a large database, there is no reason not to select the best performing MLP (MLP-1) if all seven variables are accessible, as it shows an improved sensitivity when compared to MLP-2 (0.964 versus 0.914), which means that it properly identifies COD-TC cases more consistently (although a slim overlap can be seen when looking into the 95% CIs regarding sensitivity). Furthermore, the values of MCC, PPV, NPV, and F1 score also indicate a stronger performance by MLP-1. It must be noted that even though MLP-1 was trained with a database containing less than half the number of samples than MLP-2 (8,477 vs. 20,344, respectively; Table 2), this amount is more than enough to ensure proper training and avoid over-fitting effects considering the dimensions of MLP-1, as a very high sample-to-weight ratio is maintained (further explained in Materials and Methods).

On the other hand, when comparing the performance of the MLPs with the PLS-DA models with the same inputted variables, it can be noticed that the non-linear neural network is better suited for predicting medical outcomes than the classic linear method. Although the PLS-DA classifiers are still accurate tools (Table 5), the MLPs provide stronger results for all three models, validating the use and optimization of these more powerful algorithms. Specifically, MLP-1, MLP-2, and MLP-3 presented an increase in total accuracy when compared to their PLS-DA counterparts of 7.3%, 1.8%, and 2.1%, respectively, justifying the use of ANNs for the risk assessment of thyroid cancer, especially concerning MLP-1. Finally, the other calculated metrics (MCC, PPV, NPV, and F1 score) all favour the MLP models when compared to the performance of the PLS-DA ones.

## Discussion

The ability to model tumour behaviour has large implications in the staging and prognostication of cancer. Recent advancements in the field of oncology have led to a massive expansion of clinical and genomic information that can be utilized for better understanding of the life history of a tumour. However, limitations in statistical analysis have hindered our ability to accurately understand relationships between these variables that are known to hold prognostic value, precluding their use as part of a staging system. Consequently, the method by which tumours are staged is still largely based on a system devised in 1953 by Pierre Denoix^24^. Current AJCC guidelines utilize gross clinical and pathological information to predict tumour behaviour (size, lymphatic metastases, and distant metastases). The value of this information is based on multivariate statistical analysis demonstrating prognostic impact, which is based largely in part on linear relationships between variables and does not account for partial and/or non-linear relationships or multiple co-existent states. ANNs are specifically designed to elucidate non-linear relationships, with an inherent ability to self-teach from training sets. Such algorithms are optimized to carry out image, facial, voice, and handwriting recognition, and now they have begun to be used in oncology research [^4,5^, and this work].

The increasing incidence of thyroid cancer has highlighted the need for better prognostication and understanding of tumour behaviour^25^. Through the current study, we have harnessed the power of ANNs to generate a set of models that can predict thyroid cancer survival with significantly improved accuracy. Our most accurate model, MLP-1, showed an accuracy of 94.49% (94.45% of alive cases and 96.36% of thyroid cancer related death; Table 3). Moreover, utilizing feature selection algorithms, we determined that the most useful clinical predictors of thyroid cancer are age of the patient when diagnosed, the extent of thyroid disease present (e.g. encapsulated, gross extra thyroidal extension, or pathological extra capsular extension), in addition to location of nodal disease (**MLP-2**). It is also worth noting that, due to high survivability rate of thyroid cancer, the databases used to train these MLPs are unbalanced in terms of number of data points per group to be classified (shown in Table 2; about 97.5% alive versus 2.5% COD-TC cases, respectively). Despite this fact, which typically affects the performance of MLPs, remarkable classification accuracies are achieved for both classes, signifying that strong relationships have been found between the independent and dependent variables employed^26^. In other words, variables with significant prognostic power have been identified and employed to reach reliable classifiers by our preferred models, MLP-1 and MLP-2. On the other hand, MLP-3, which was generated based on the TNM tumour staging system was not able to predict survival (80.84%) nor death (81.40%) at the same rate as the other models, as its global accuracy is 13.6% and 10.2% lower than MLP-1 and MLP-2, respectively (Table 3), also supporting current limitations in thyroid cancer modelling based on AJCC guidelines^15^. It is worth mentioning that in the present research, a classification problem has been carried out to serve as a prognosis assessment. This is not the typical methodology employed for such studies, which are usually evaluated via Cox proportional hazards analysis^27^.

Regarding the identified variables with the highest prognostic value for thyroid cancer (via feature selection), ***age*** was first described as such by Byar *et al*. in 1979^28^. Since then, multiple studies and indices have employed age as an important component when staging and predicting disease behaviour in thyroid cancer, including the Mayo Clinic’s MACIS index and the Lahey Clinic’s AMES index^13,29,30^. The AJCC *Cancer Staging Manual* has utilized age since its 3^rd^ Edition, based on a 55-year old cut-off^31^, and since then, large-scale retrospective studies have reinforced its prognostic role^29^. In 2015, Ganly *et al*. recognized age as a predictive variable and established a nomogram using regression analysis to predict survival^32^, promoting the use of age as a continuous variable. As age increases, the prognosis declines, however, this may not necessarily correlate in a linear relationship, especially when considering its combination with other clinical factors (e.g. with the presence of lymph node metastases or a tumour with gross extracapsular extension). By utilizing ANNs, the inter-variable relationships and their influence on prognosis can be handled by giving a weighted impact of inputs and their combinations. This allows age to be a dynamic influencer on prognosis that may change from patient to patient and be affected by the presence of differences in other variables, as opposed to nomograms that only identify static influences. This notion is epitomized by the new AJCC staging system that acknowledges the changing impact of age in patients over the age of 55, especially when determining the prognostic role of lymph node location^31^. Our model accurately predicts this ideal, but without using cut-offs, allowing for age to be dynamic and continuous in its impact.

The 2015 American Thyroid Association’s guidelines determined lymph node number, size, and presence of extranodal extension as being prognostic drivers in impacting risk of persistent/recurrent disease, whereas some studies, including the most recent AJCC 8^th^ Edition guidelines, disregard ***location of lymph nodes*** as impacting prognosis in patients younger than 55^25,31,33^. Recently, in 2017, Sapuppo *et al*. did however demonstrate that lymph node status was the best prognostic factor in predicting thyroid cancer-related death for particular kinds of thyroid cancer^34,35^. Our devised MLP-2 and FS algorithms support the conclusion by Sapuppo *et al*., finding a high predictive value of lymph node location.

Lastly, the ***extent of primary disease*** has also been recognized as having prognostic value in thyroid cancer^36^. In 2010, Baek *et al*. found that extrathyroidal extension was correlated with recurrent cervical neck disease^37^. Riemann *et al*., in 2010, demonstrated an improvement in disease free events in patients with minimal extrathyroidal extension when compared to those patients with sizable extrathyroidal extension^38^. Ito *et al*. utilized univariate statistical analysis to determine that massive extrathyroidal extension decreased relapse free survival when compared to minimal extension^39^. Consequently, the amount of primary disease extension has been incorporated in the AJCC cancer staging manual^31^. The aforementioned studies however are limited in determining the exact impact of disease extent on prognosis by utilizing univariate and multivariate statistical methods that do not demonstrate a dynamic relationship with other variables. Our MLP-1 and MLP-2 have validated the relevance of extrathyroidal spread leading to more accurate prognostic modelling by allowing it to have variable weighting depending on the value of other clinical variables, most notably age and location of positive nodes.

By applying the FS algorithms to the seven variables used for MLP-1, the three aforementioned variables were identified as the ones with the greatest prognostic power and used to train MLP-2. The prediction of thyroid cancer outcomes was still possible, while maintaining a strong statistical performance in terms of global accuracy (from 94.5% (MLP-1) to 91.1% (MLP-2); Table 3), although the correct estimation of COD-TC cases (sensitivity) was slightly lower (from 96.4% to 91.4%; Table 3). Therefore, MLP-2 showcases the power of ANNs as they were able to correlate primary disease extension, inherent within its algorithmic design, without having to directly link, for example, with size dimension of the primary tumour, which was removed after the FS process. Furthermore, an accurate prognosis predicting system with only three required variables should be highly beneficial for both patients and physicians.

Through our research we were able to utilize artificial intelligence to predict thyroid cancer patient survival and related deaths. However, given the mostly indolent nature and high percentage of survival of thyroid cancer patients, the standard has shifted from predicting survival to predicting risk of recurrence^25^. The currently prevailing staging method, TNM, has an inherent shortcoming in predicting recurrence as is known in the field. Moreover, and unfortunately, the SEER database does not include status on cancer recurrence. Large-scale recurrent data would allow for a more clinically useful ANN to be derived that could be used to predict disease recurrence instead of survival. Hopefully, our results will prompt others to include medically important features such as recurrence when building their future patient database, as ANNs provide an invaluable method by which to utilize oncological data, enabling forthcoming research that can incorporate diverse types and large amounts of data. Machine learning can be employed for much more beyond the incorporation of clinical data as we proposed, including mining and utilizing genomic data, a current focus of thyroid research as well as of many other medical fields^40^.

ANNs are exciting algorithmic tools that allow for an improved modelling of variable relationships that can be applied to cancer prediction research. We were able to design, train, and optimize a 3 variable ANN (MLP-2) that was able to predict thyroid cancer outcome accurately. The attained 91.1% of correct classifications represents a ∼10% increase in accuracy when compared to traditional TNM (also 3 variables; Table 3) tumour staging methodology (MLP-3). Furthermore, these classifications showed an enhanced performance when compared to the results provided by a more classic modelling approach such as PLS-DA.

Nevertheless, it is relevant to note that the present study is limited to the analysis of a single thyroid cancer-related SEER database, not considering any other data source or omics derived information. Future algorithms could benefit from the inclusion of, for instance, data collected from genomics, proteomics, or metabolomics studies. Furthermore, the presented MLP models would improve and become more generalizable if successfully validated or even reoptimized with data from multiple sources and/or populations combined.

As final remarks, we have shown that models based on MLPs can be used to interpret and extract underlying relationships between clinical variables and a thyroid cancer patient’s outcome or prognosis. Straightforward databases from unspecialized and existing medical records have been converted into cognitive algorithmic tools that can reliably estimate a vital characteristic such as disease prognosis, which can guide doctors towards informed and optimized treatment decisions. In the future, the principle behind our machine learning approach can be implemented to predict, during the design of clinical trials, the likelihood of beneficial effects among certain subpopulations representing certain traits, while minimizing the risks associated with others when testing new drug candidates.

## Materials and Methods

### Database

The database for the study was obtained from the November 2014 submission of the U.S. SEER-18 database^41^. A cohort of thyroid cancer cases was created by the International Classification of Diseases for Oncology, 3^rd^ Edition (ICD-3). The data was restricted to the select histologic subtypes papillary carcinoma and follicular carcinoma. Only thyroid cancer cases diagnosed between 1988 and 2007 were included to allow for adequate follow up, leading to a total of 61,362 data entry points. The database excluded data from Louisiana during the periods of Hurricanes Katrina and Rita from July to December 2005.

Demographic data on date of diagnosis, patient age, gender, and race were obtained. Surgery type was categorized into total thyroidectomy, subtotal thyroidectomy, lobectomy, and no surgery. Radiation was classified as beam radiation, radioactive isotope, combination of beam and implant or radioactive isotope, other (radiation not otherwise specified, radioactive implants), and none. Both number and location of lymph nodes were subclassified as none, regional, distant, and unknown for the purpose of analysis. Extent of disease was then examined and classified into *in-situ*/no evidence of primary disease, intrathyroidal spread, pathological extrathyroidal spread, gross extrathyroidal spread, metastasis, and unknown. Size of the primary tumour was also stored as pathological size. This information led to a database containing 34 clinical variables (e.g. age, cancer grade, radiation in relation to surgery, primary tumour size, regional nodes examined, survived months, and so on) which were all analysed, and several employed as independent variables in the modelling phase.

### Data arrangement and pruning

In first place, as many of the clinical parameters present in the database were categorical, they were transformed into mathematical variables by labelling each class within a parameter accordingly (e.g. for the gender variable, “0 s” were assigned to males and “1 s” to females). Then, an initial reduction of the number of samples, guided by the end goal of this research, took place. In this first pruning stage, only the information from patients which were still alive or had died due to thyroid cancer were kept, leading to a decrease from 61,362 to 57,157 samples (55,437 alive cases and 1,720 “cause of death-thyroid cancer” (COD-TC) cases; this pre-processed dataset is shown in the Supplementary Information section (Excel sheet: “Database of Variables Used”)). Afterwards, to further ascertain the purpose of the novel mathematical models (MLP-1 and MLP-2), the samples were limited to people that have been alive for over ten years since the diagnosis, and people that passed away within the first five years, leading to a database containing 25,063 entries (24,025 alive cases and 1,038 COD-TC cases) (Table 1).

### ANNs used and feature selection

The ANNs employed are MLPs, which are composed of several layers, covering from input data to output information, in an end-to-end estimation mode. An input layer defined by a set of independent variables (or nodes) that are used to train the network. The second layer is a hidden layer, which is formed by artificial neurons where the bulk of the calculations take place. The final layer is the output layer and consists of the dependent variables that the network is trained to predict, and also is formed by artificial neurons (as many as dependent variables; only one for each of the three models in this study, as they are binary classifiers (Table 2)). MLPs are trained with a set of known independent and dependent variables, to “teach” the network the desired outcome based on the inputs. Through iterative calculations, the network will learn to model the dynamic interactions of the variables^16^.

Three different MLP models were created and the captured clinical variables were used as input. The first network (MLP-1; Fig. 1) utilized seven clinical variables including age, race, gender, tumour size, number and location of positive lymph nodes, and primary disease extent, which were taken from the original 34 based on findings in the literature^13,24,28–39,42,43^. Subsequently, three different filter-based FS algorithms (Fisher’s discriminant ratio^20^, Kruskal-Wallis’ analysis^21^, and Relief-F algorithm^22^), based on unique mathematical criteria, were independently tested on the seven variables to locate the three most predictive variables that were utilized to create a second MLP (MLP-2; Fig. 3). Only three variables were selected to reach an architecture that is comparable to MLP-3. The overall performance of the variables for the three methods was analysed to reach an informed selection. Filter-based FS algorithms analyse the variables individually and rank them according to their discriminative power for a successive task. These methods do not consider potential redundant information that different variables may possess, reason why they are mainly used as a fast pre-processing tool^44^. Finally, utilizing the AJCC tumour staging guidelines, a third network (MLP-3; Fig. 4) was created using tumour size (T), nodal status (N), and presence of metastases (M) as three predictive clinical variables.

### ANNs – training and optimizing MLPs

MLPs are the most employed type of ANN^45^, and as any supervised model, they require each data point to be labelled (“0 s” for alive cases and “1 s” for COD-TC cases). Inside every MLP there is a set of weighted parameters (or weights) that connect every unit (nodes and neurons) from one layer with all units in neighbouring layers. These weights are initially given a random number (between 0 and 1), and during the training process, they are modified to lower the error of the MLP (increase the patient classification accuracy). Therefore, training a MLP can be understood as optimizing the weights during the learning phase of the model. In this phase, the database is divided randomly into two datasets, namely training and verification. The MLP uses the training set to modify the weights, and the verification set to evaluate the performance of the model intrinsically with data not employed to change these weights. In other words, the verification dataset is a group of samples that the MLP utilizes to ensure it avoids overfitting for the training dataset and is able to generalize for external data^45^.

Besides the weights, other parameters also have to be optimized or selected before reaching an optimized MLP. They are the training and transfer functions, the number of hidden neurons (NHNs), and the learning coefficients. The training function embodies the equation that is in charge of the weight modification. In this study, the Levenberg-Marquardt backpropagation has been implemented, as it is the quickest training algorithm for moderate-sized MLPs, possessing a memory reduction feature for large training datasets^46^. The transfer function restricts the range of the values given by every neuron. In this case, the non-linear sigmoid function has been employed, which limits data between 0 and 1^45^.

Another crucial parameter is the NHNs. These hidden neurons must be optimized adequately as MLPs with a low NHN may have a hampered learning capability, and, therefore, may not be able to fully interpret the non-linear relations between variables, resulting in inaccurate models^47^. On the other hand, a high NHN could lead to overfit systems that are not able to generalize well for data that is external to the learning dataset. A heuristic method has been employed to optimize the NHNs, testing all possibilities within a logical window that would never lead to models with NHNs lower than 3 or less than a 50-to-1 sample-to-weight ratio (to avoid overfitting)^45^.

Finally, an adequate combination of the learning coefficient or Marquardt adjustment parameter (Lc), and its decrease (Lcd) and increase (Lci) factors, has to be used in the MLPs. The Lc embodies the learning coefficient in classic backpropagation algorithms^48^, and it is decreased and increased by Lcd and Lci, respectively, until the changes lead to a deteriorated statistical performance. The evaluated values ranged from 0.001 to 1 for Lc and Lcd, and from 2 to 100 for Lci^48^.

### Validating the MLPs

Independent testing, which utilizes “blind” samples to determine the performance and generalization capability of the MLPs, has been performed. For this process, the databases are randomly divided into three: training, verification, and test (“blind”) datasets, containing approximately 70%, 20%, and 10% of the samples, respectively. Furthermore, this process was carried out three times for each model to ensure robustness and flexibility (three different random divisions of data) and the final reported statistical performances result from the averages of these three tests (Table 3)^45^. Also, ROC curves for each classifier have been depicted for further evaluation (Fig. 2). The AUCs of ROC curves are proportional to the performance of the classifiers, where an AUC of 1 means 100% accuracy (best performance) and of 0.5 signifies absolute random classification^49^.

### PLS-DA for Comparison with MLPs

As a final step, in order to justify the use of machine learning-based algorithms to carry out this classifying task, PLS-DA models have been calculated for statistical comparison. PLS-DA is a classic mathematical approach based on creating linear regressions to estimate categorical variables. Three PLS-DA models have been calculated using the same independent variables and datasets as MLP-1, MLP-2, and MLP-3.

All calculations performed for this manuscript have been completed via MATLAB version 9.3.0.713579 (R2017b)^16^.

## Supplementary information

Dataset 1.

## Data Availability

The database analysed during the present research is available from the corresponding author upon request.
